# Bridging the Gap between the Technological Singularity and Medicine: Highlighting a Course on Technology and the Future of Medicine 

**DOI:** 10.5539/gjhs.v5n6p112

**Published:** 2013-09-09

**Authors:** Kim Solez, Ashlyn Bernier, Joel Crichton, Heather Graves, Preeti Kuttikat, Ross Lockwood, William F. Marovitz, Damon Monroe, Mark Pallen, Shawna Pandya, David Pearce, Abdullah Saleh, Neelam Sandhu, Consolato Sergi, Jack Tuszynski, Earle Waugh, Jonathan White, Julielynn Wong, Michael Woodside, Roger Wyndham, Osmar Zaiane, David Zakus

**Affiliations:** 1 Faculty of Medicine and Dentistry, University of Alberta, Edmonton, Alberta, Canada; 2 Faculty of Business, University of Alberta, Edmonton, Alberta, Canada; 3 Faculty of Arts, University of Alberta, Edmonton, Alberta, Canada; 4 Faculty of Science, University of Alberta, Edmonton, Alberta, Canada; 5 MDM, 415 Laurelwood Ln, Southbury, CT 06488, USA; 6 Microbial Genomics Section, Division of Microbiology and Infection, Warwick Medical School, University of Warwick, Coventry CV4 7AL, UK; 7 BLTC Research, 7 Lower Rock Gardens, Brighton BN2 1PG, UK; 8 Center for Innovative Technologies and Public Health, Toronto, Ontario, Canada; 9 Department of Renal Medicine, Concord Repatriation General Hospital, Hospital Road, Concord NSW 2137, Australia

**Keywords:** technological singularity, artificial intelligence, nanotechnology, quantum biology, future of medicine course, medicine writ large, Rudolf Virchow, global health

## Abstract

The “technological singularity” is forecasted to occur in the mid-21^st^ century and is defined as the point when machines will become smarter than humans and thus trigger the merging of humans and machines. It is hypothesized that this will have a profound influence on medicine and population health. This paper describes a new course entitled “Technology and the Future of Medicine” developed by a multi-disciplinary group of experts. The course began as a continuing medical education course and then transitioned to an accredited graduate-level course. We describe the philosophy of the course and the innovative solutions to the barriers that were encountered, with a focus on YouTube audience retention analytics. Our experience may provide a useful template for others.

## 1. Introduction

The *technological singularity* is the projected convergence of humans and machines. This is predicted to occur around the mid-21^st^ century when machines outsmart their human creators (Kurzweil, 2005). The singularity concept is now entering mainstream discourse as shown by Time Magazine’s February 2011 cover entitled “The Singularity - 2045 - The Year Man Becomes Immortal” (Grossman, 2011) and the November 2012 announcement that iPad and iPhone assembler FoxConn is replacing one million human workers with one million robots (Hill, 2012). While readers will be aware of both of these events, they may not fully appreciate their true long-term strategic importance.

Although the singularity could impact all facets of medicine, thus far only a single PubMed indexed article references the term “technological singularity”, a 2010 review on the future of high throughput sequencing in clinical microbiology (Pallen, Loman, & Penn, 2010). The National Library of Medicine does not list “singularity” as a MeSH term.

The *technological singularity* is the additive sum of innumerable technological advances that in aggregate could lead to a rupture in the fabric of human history that will have completely unpredictable results. It could lead to a utopian post-scarcity world in which disease has been essentially eliminated, and human beings have everything they want and need (Diamandis, 2012). Or it could lead to enormous catastrophic global suffering and loss of life and possibly mark the end of humanity (Tonn & Stiefel, 2013). With knowledge and foresight, it may be possible to shift the impact of the *technological singularity* to a positive outcome. This is one of the long-term objectives of our course.

For 2 years, a group of dedicated faculty has been teaching a unique, multidisciplinary course at the University of Alberta that addresses the issues *of the impact of technology and the technological singularity on the future of medicine*. It is open to students from all backgrounds. Guest faculty from around the world lecture via video teleconferencing using Skype or similar platforms. The lecture videos are available on YouTube at http://www.youtube.com/user/kimsolez. A Facebook group was established at https://www.facebook.com/groups/223876451014842/ to share relevant information about technology, medicine and the technological singularity. Lecture topics are listed in Table 1.

**Table 1:** Representative list of “Technology and the Future of Medicine” course lectures at the University of Alberta and Number of Views on the YouTube channel http://www.youtube.com/user/kimsolez as of August 10, 2013 (in chronological order where multiple videos exist, lectures at bottom do not have videos yet)

Introduction/The Future of Medicine (634, 484)
The Technological Singularity Explained and Promoted (129, 482)
Promise and Perils of Artificial Intelligence (x4) (170, 60, 32, 270, 138)
Evil As A Treatable Disease (131, 148)
Promise and Perils of Nanotech (x2) (1776, 766)
Whales, Robots and You: How Technology May Change What You Think Of As A Person (120)
Medical Ethics in a World of Robots (93, 116)
General Artificial Intelligence, The Singularity Inside & Out (http://www.hutter1.net/publ/sasingularity.pptx)
Quantum Biology (997)
Will Humanity’s Successors Be Our Descendants? (Teaching session is mainly discussion via Skype) (208)
FutureHype (56) The Elixir of Life: Magic, Technology, and Medicine (298)
Is Technology Making Us Fat? (116)
The Singularity and The Have Nots (The example of Nepal) (326, 67, 256) (Solez et al., 2012)
Entrepreneurship in Medicine/Innovation (98, 89, 77)
Privacy of Medical Information in the Future (83)
Entrepreneurship in Medicine Workshop (121, 66)
Technology and Global Citizenship (135, 61)
Artificial Intelligence and Games (106)
Technology and the Body (1496, 152, 268, 639, 320)
Writing as a Technology (39)
The Singularity in an Industry Context (41, 164)
Atomic Force Microscopy (128)
From Sense to Intelligence: Enhancement of the Human Mind (512)
Promise and Perils of Biotech
There’s Still Plenty of Room at the Bottom
3D Printing and Medicine
Use of Social Media in Medicine

Although the topics of artificial intelligence, nanotechnology and quantum biology may not directly impact the practice of medicine today, they are very relevant to the medicine of tomorrow and to the medicine writ large concept (Solez, 2013). This is an idea first advanced by the father of cellular pathology, Rudolf Virchow. He stated that medicine encompasses not only disease recognition and treatment but also physical and spiritual human enhancement and improvements in society to promote health and wellbeing (Virchow, 1848).

Lectures on the *technological singularity*, artificial intelligence, nanotechnology, and medical ethics are scheduled first so the student obtains a fundamental knowledge base before more specialized subjects are added. A balance is maintained between technological advocacy and skepticism and between scientific and humanistic approaches. A global health perspective is also included through lectures that discuss the impact of technology on marginalized and vulnerable populations, and the idea that beneficial new technologies should be of service to all.

## 2. History of the Creation of the Technology and Future of Medicine Course

The course was conceptualized in the spring of 2011. It was presented to undergraduate and graduate student focus groups in May 2011. Several key recommendations emerged from these focus groups and became part of the final course plan. The course was designed to be interdisciplinary to a degree not observed before with student and faculty input from business, law, engineering, science, art, medicine, global health, and other disciplines. It was made available to the public and students in all faculties. The consensus was that the course should also be available to undergraduates who were judged by an interview process to have the requisite maturity to participate fully in course discussions. Each student would select a faculty mentor, identify a relevant area of special interest and prepare a paper and presentation, which would form the bulk of the student’s grade. Students would be encouraged to contemplate and discuss positive and negative projected individual and societal changes and disruptions caused by the technological singularity.

The course was formally proposed in September 2011. It was first taught as a continuing medical education (continuous professional learning) course in the fall of 2011. The course was launched as a regular university graduate course in the winter of 2012. It is now taught every fall and winter term. In the winter of 2012, there were two graduate students taking the course for a grade, accompanied by twelve continuous professional learning students. The two graded students became strong advocates for the course. This led to five students registering the next term and then fourteen in the following semester.

## 3. Course Description

There are twenty-six 80-minute teaching sessions per semester. Each course includes two student presentation evenings, one midway into the course, and one at the end. A special guest faculty presentation is also included on one of these two presentation evenings. Associated social events facilitate discussion and interaction. See http://www.singularitycourse.com for the course website.

A traditional interdisciplinary course usually focuses on a fixed theme and urges scholars from diverse methodological approaches to provide their analysis of that theme. In our course, a variety of viewpoints are expressed by recognized experts from different fields which allows students to view the medical dimensions of technology through different lenses. Much of traditional scholarship is based on an implicit confidence in the validity of the past in organizing and predicting the future. Once you remove this past, present and future connectedness structure, you undermine most of what scholarship has been built upon. By moving beyond this model, our course facilitates creative interaction, unorthodox thinking, and even ideological clashes.

## 4. Discussion of a Major Theme of the Course: Quantum Biology

One course topic, quantum biology, is less familiar and deserves further explanation here.

### 4.1. Quantum Biology

Both physics and chemistry depend on the power of quantum mechanics to provide fundamental insights into the world around us. At present, the known quantum effects only play a minor role in biological processes, which mirrors the state of quantum mechanics in the field of physics one hundred years ago.

Below, we provide a brief overview of efforts to apply quantum principles to biology.

Fritz Popp and his collaborators (1981) demonstrated that photons, or electromagnetic energy quanta can be absorbed and emitted by DNA molecules and this involves low-intensity ultraviolet ranges of the spectrum. Guenter Albrecht-Buehler (1992) demonstrated experimentally that living cells perceive infrared electromagnetic waves with a sensitivity peak around wavelengths of 1000 nm. He hypothesized that mitochondria are involved in energy production through a proton transfer mechanism. Centrioles are intricately structured to absorb these photons and trigger a signaling cascade.

Hecht, Schlaer and Pirenne (1942) showed that the human eye is capable of detecting light at the resolution of 2-3 photons. Luca Turin (2002) and Marshall Stoneham and his collaborators (2007) have provided strong arguments that the sense of smell is based on a quantum resonant energy transfer mechanism involving vibrational degrees of freedom of aromatic molecules and receptors in the membranes of olfactory nerves.

Herbert Froehlich (1968) postulated that quantum coherence is an inherent property of living cells, which utilize it for long-range interaction purposes such as synchronization of cell division processes. However, only scant experimental evidence exists to support these claims (Webb 1980; Grundler & Keilman, 1983). Attention has recently been brought to the quantum mechanical nature of photosynthesis (Arndt, Juffman, & Vendral, 2009; van Grondelle & Novoderezhkin, 2006) where reaction centers capture individual photons and transfer exciton energy by tunneling and thereby avoiding decoherence at room temperatures. Klaus Schulten (Hu, Ritz, Damjanovic, Autenrieth, & Schulten, 2002) has advocated the use of quantum mechanics by purple bacteria and light harvesting in carotenoids. He demonstrated the feasibility of quantum effects by performing high performance computing at the atomic level of simulation for the relevant enzymatic reactions. This is a natural progression of theory development which was preceded by extensive experimental (Möbius & Kuhn, 1979) and theoretical (Tuszynski, Joergensen, & Möbius, 1999) work on the so-called Scheibe aggregates. The term refers to thin films composed of chromophore molecules that have been engineered to harvest light energy and funnel the quanta of exciton energy at specific sites through quantum hopping and funneling. Klaus Schulten and his collaborators (1978) hypothesized some 25 years ago and other groups (W. Wiltschko & R. Wiltschko, 2005) have recently demonstrated that bird navigation is based on quantum entanglement that persists for at least 20 microseconds which is longer than the currently maintained laboratory experiments at comparable temperatures.

Johnjoe McFadden (2000) maintains that the process of biological evolution in bacterial mutations under toxic stress must involve quantum computing in order to explain its efficiency and anticipatory steps. Quantum theories of consciousness have been proposed by Penrose and Hameroff (1996) but their highly controversial assumptions have been hotly debated (Abott, Davies, & Pati, 2008). A general theory called quantum metabolism (Demetrius & Tuszynski, 2010) states that metabolic energy is universally generated by all living systems using the principles of quantum mechanics. This theory has now been extended both to cancer (Davies, Demetrius, & Tuszynski, 2012) and neurological diseases like Alzheimer’s (Demetrius & Simon, 2013).

In summary the growing field of quantum biology promises to invigorate the debate about the conceptual foundations of biological processes and also provide major implications for the future of medicine with such concepts as coherence and long-range order providing a new perspective on human health.

## 5. YouTube Course Videos

We record broadcast quality video and audio of each teaching session using three high-end cameras and high-quality wireless microphones. An important course innovation has been the uploading of videos on YouTube, each with a table of contents with clickable hot links at (http://www.youtube.com/user/kimsolez). These hot links permit viewers to jump immediately to their areas of interest. We discovered that the indexed 20 minute discussion period at the end of each lecture is often the most viewed aspect.

## 6. Course Feedback

Students evaluate each lecturer and make recommendations via anonymous SurveyMonkey forms. These critiques rate how well particular lecturer’s material fit into the overarching themes of the course. This feedback is used to make changes to the lectures mid-course and in subsequent semesters.

## 7. Methods for YouTube Analytics

YouTube analytics of the course videos are also used to derive feedback. The detailed hot-linked table of contents in the YouTube descriptions of the course videos allows viewers to return precisely to their favorite portions of the videos and watch those parts repeatedly if they desire. It is also possible for them to recommend that others watch select portions based on the tables of contents. This results in clearly identifiable peaks in audience retention corresponding with specific slides and content in the video. This is highly informative in identifying those parts of the lecture that had the greatest impact. The analysis does not distinguish multiple views by different people from multiple views by one person.

YouTube audience retention analytics were performed between May 29th and June 8th 2013 on all course videos on lifetime audience retention and audience retention over the past 30 days. The basic YouTube statistics can be accessed by clicking the chart “statistics” icon just to the lower right of the video just below the number in the number of views. View numbers were also examined for each video on August 10, 2013.

## 8. Results of YouTube Audience Retention Analytics

Five prominent audience retention peaks were noted. They identify moments in the course videos that audiences particularly connected with. Often these peaks correspond to statements about the imagined future and evocative imagery about what the future will be like.

**Figure 1:**
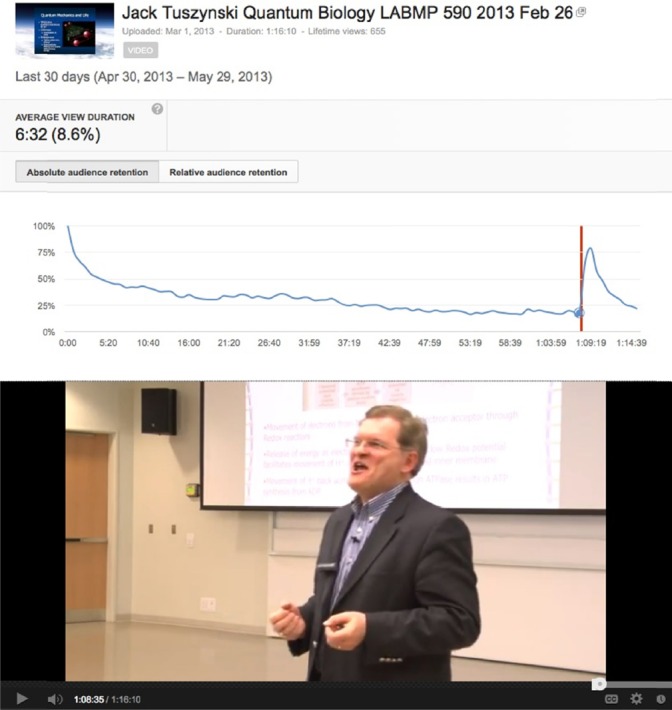
Audience retention peak corresponding to “All the textbooks will be rewritten” statement in Quantum Biology lecture by Jack Tuszynski

The most dramatic example of an audience retention peak occurs in the “Quantum Biology” lecture by Jack Tuszynski at one hour eight minutes and 30 seconds (Figure 1). At this point, the lecturer states, “It is a revisionist approach to biology. Probably all the textbooks will be rewritten by quantum physicists, with every example from actin to microtubules to centrioles to neurons being rewritten as a quantum phenomenon. Of course at the end remember classical physics was not thrown out. It’s not garbage. It is still useful, but you have to keep it at a certain level. So classical physics is useful at the level of designing trains and looking at ping pong balls. But if you look at the fundamental nature of matter, you need to use quantum mechanics, the same with biology. You can still keep your cartoonish visions of how cells operate, but if you really want to understand how mitochondria work, how various enzymes operate, how recognition operates at a subcellular level, you have to go to quantum mechanics.”

**Figure 2:**
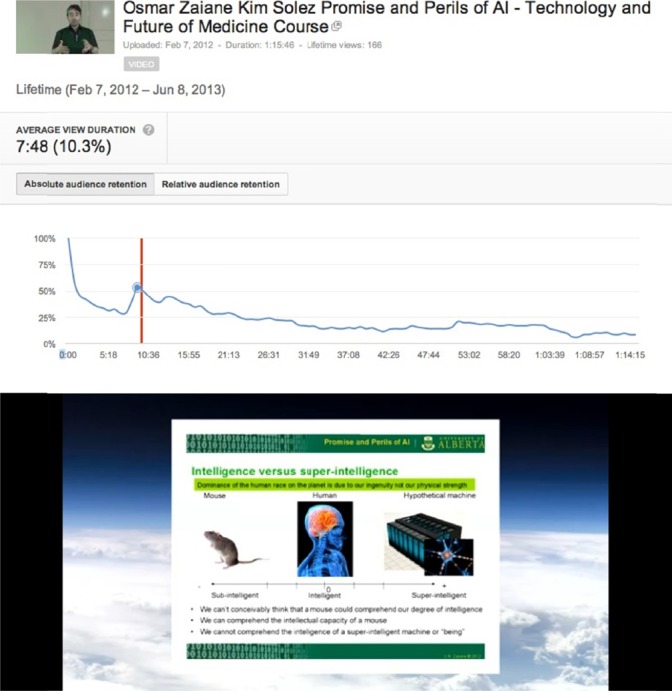
Audience retention peak corresponding to description of the intellectual life of a mouse and intelligence versus super-intelligence from the first lecture on Artificial Intelligence by Osmar Zaiane

Another example is the description of the intellectual life of a mouse versus a human, and intelligence versus super-intelligence from the first lecture on Artificial Intelligence by Osmar Zaiane, see Figure 2.

A third example is the student discussion of the FRET technique of studying protein interactions using fluorescence (Lle`res et al, 2007; Pistor and Kremeers, 2007) from the Entrepreneurship in Medicine Workshop by Shawna Pandya.

**Figure 3:**
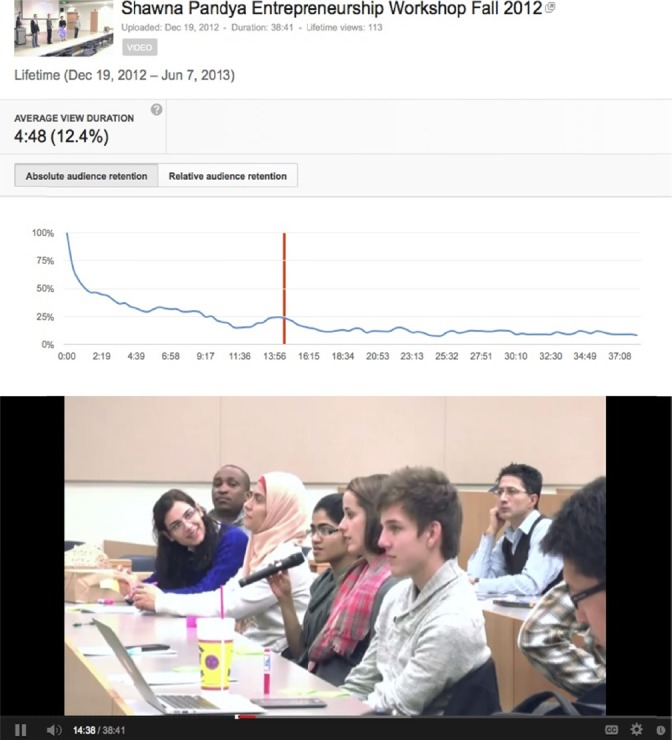
Audience retention peak corresponding to student discussion of the FRET technique of studying protein interactions using fluorescence from Entrepreneurship in Medicine Workshop by Shawna Pandya

A fourth example from the same workshop in the Winter Term 2013 shows the presentation by one of the student teams describing their new company and its main product.

**Figure 4:**
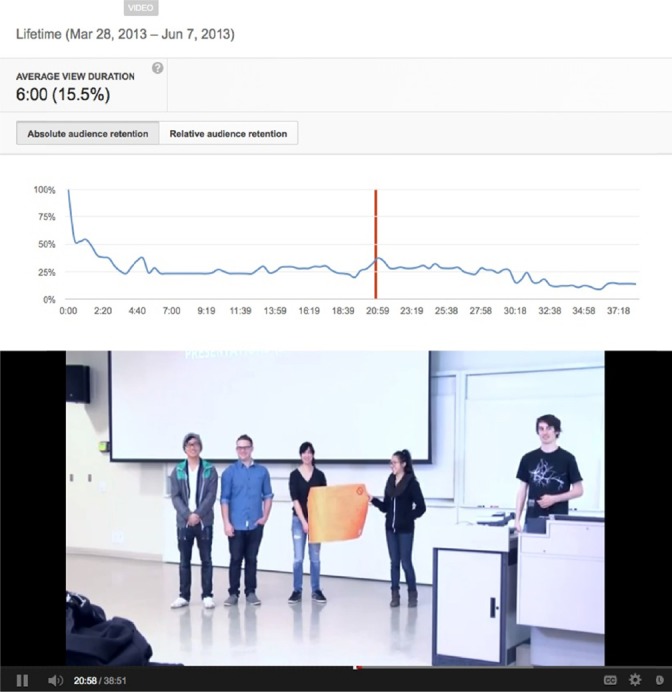
Audience retention peak corresponding to student team from the Entrepreneurship Workshop describing their new company “Selective Telecommunications For Us” and its main product the Subtransmuter 1000

A fifth example is the reference to the flying car in the Entrepreneurship in Medicine/Engineering the Future lecture by Shawna Pandya. The car is expected to be production ready in 2015.

**Figure 5:**
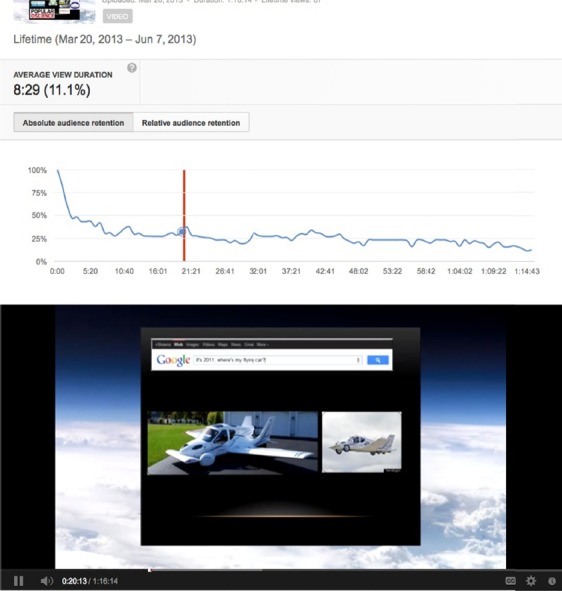
Audience retention peak from the reference to the Terrafugia flying car in the Entrepreneurship in Medicine/Engineering the Future lecture by Shawna Pandya

## 9. Results - Number of Views for YouTube Course Videos

In the 2013 winter semester course, there were a total of seventeen students. Of these, fourteen were graded and three were auditing the course for professional development. The most viewed video is Michael Woodside’s “Nanotechnology Part I” from January 2013, which had over 1700 views as of August 10, 2013. The ratio of in-class versus online viewers of the lectures was approximately 1:100. The second most watched course video is Jonathan White’s “A Biological Repairman’s Reflections on the Coming Singularity: Notions of Embodiment in the Age of Spiritual Machine” from the fall of 2011 which garnered 1,496 views as of August 10, 2013.

For comparison, the Singularity Summit 2012 videos maxed out at about 5,000 views and past Singularity Summit videos achieved a total of about 33,000 views. Ray Kurzweil’s original “Technology Will Transform Us” TED talk from February 2005 has had 1.2 million views (TED, 2013). The TV situation comedy show “The Big Bang Theory” program that mentions the technological singularity and related subjects reaches 20 million viewers in the US alone (CBS, 2013). The PBS Nova program that discusses similar subjects seriously reaches 7 million viewers worldwide. PBS itself reaches 124 million viewers (PBS, 2011).

## 10. Discussion

The *technological singularity* is an event that may happen in the future as the consequence of diverse, exponentially accelerating innovations. One needs to be prepared for it to be able to positively harness its potential. There is well-developed theory and research on the diffusion of innovations (Rogers, 2003). Our “Technology and Future of Medicine” course represents one strategy in the dissemination of the concept and implications of the *technological singularity*. A course embracing one particular dogmatic idea about the future is of limited value. Its predictions may be entirely inaccurate. However, a futures studies course like ours in which a number of different vantage points and possible future scenarios is presented is of much greater value.

One of the more interesting features of the course is the way in which it caused the *students* to make predictions about the future based on lecturer’s insights. One excellent example of this occurred in UK philosopher David Pearce’s (Ford, 2013) teaching session wherein students saw him speak for about ten minutes in a video and then conversed with him directly via Skype for the remaining 70 minutes. This outcome – having students extensively explore their own predictions of the future - demonstrates the validity of the course’s design as a mechanism of learning. It shows that the students grasp the material when it is presented this way and are motivated to make contributions to the various possibilities by offering their own insights and coming to a mutual understanding (Rogers, 2003). This David Pearce session and others like it constituted our version of “flipping”, that is reversal of the traditional education model, with students accessing the content during their own time and then using class time for discussion and answering questions (Waldrop, 2013).

Diversity and balance in presentation, incorporation of technology skeptics as well as technology advocates in teaching as we have in our course, is crucial to the dissemination of these ideas. When one compares the 7 million people reached by the PBS Nova program as the potential audience and the 1700 views the course videos reach currently, the challenge of expanding the audience is obvious.

Another approach to further dissemination of these ideas is the creation of more courses. The authors hope that other courses on technology and the future may spring up in universities elsewhere and that this paper is of value to others who would like to emulate our course incorporating their own special local flavor.

Our method of applying analytics to the hot-linked tables of contents of our YouTube course videos could spawn a new field of learning informatics. This “big data” approach to education may uncover new insights into the teaching process (Waldrop, 2013). The process can begin to sound very mechanical and impersonal but is actually capable of producing better one-on-one educational interactions than have ever occurred before (Norvig, 2013). Our analysis informed us that some of the most memorable parts of the course are the personal vignettes told by the lecturers. It appears that these vignettes allow the students to empathize and better connect with the lecturers.

The incorporation of broadcast quality YouTube videos in our course appears to be part of a growing trend in scientific research and education. The high impact journal Nature has recently incorporated a YouTube adjunct to some of its articles (Thelwall et al., 2012). The journal PLoS ONE has recently published an 8-page scholarly article analyzing the scientific impact of TED Talks YouTube videos (Sugimoto et al, 2013). In August 2013 Scientific American and Nature collaborated on a special report on education through online videos entitled, “Learning in the Digital Age” (The Editors, 2013). Research on use of YouTube in medicine has some analogy with research on use of other social media tools in medicine such as Facebook (George 2011; George et al, 2013; White et al., 2013).

In summary, this paper describes an innovative course in a rapidly evolving field, delivered by a highly diverse group of experts. The course provides a forum for multidisciplinary discussion of diverse subjects related to technology and the future of medicine. We hope that that the description provided herein will promote curriculum reform and encourage others to create similar courses. Beyond that, we hope that establishment of this educational model will increase the likelihood of a positive outcome for humanity in the technological singularity’s predicted coevolution and convergence of humans and machines.
